# Using large language models to automate summarization of CT simulation orders in radiation oncology

**DOI:** 10.1002/acm2.70310

**Published:** 2025-10-27

**Authors:** Meiyun Cao, Shaw Hu, Jason Sharp, Edward Lee Clouser, Jason M. Holmes, Linda L Lam, Xiaoning Ding, Diego Santos Toesca, Brianne C Raulston, Wendy S Lindholm, Samir H. Patel, Sujay A Vora, Peilong Wang, Wei Liu

**Affiliations:** ^1^ Department of Radiation Oncology Mayo Clinic Hospital Phoenix Arizona USA; ^2^ Corcoran College of Arts and Sciences George Washington University‐Washington DC Washington DC USA

**Keywords:** atomization, CT simulation order, large language model, radiation oncology, summary

## Abstract

**Background:**

In the current clinical workflow of radiation oncology, therapists manually summarize the physician‐issued Computed Tomography (CT) simulation orders to prepare patients' CT simulations. This process increases the workload, introduces variability in documentation quality, and is prone to human errors.

**Purpose:**

This study aims to address these challenges using a large language model (LLM) to automate the generation of summaries from the CT simulation orders and evaluate its performance.

**Methods:**

A total of 607 CT simulation orders were collected from the Aria database at our institution. A locally hosted LLaMa 3.1 405B model, accessed via the Application Programming Interface (API) service, was used to extract keywords from the CT simulation orders and generate summaries. The downloaded CT simulation orders were categorized into seven groups based on treatment modalities and disease sites. For each group, a customized instruction prompt was developed collaboratively with therapists to guide the LLaMa 3.1 405B model in generating summaries. The ground truth for the corresponding summaries was manually derived by carefully reviewing each CT simulation order and subsequently verified by therapists. The accuracy of the LLM‐generated summaries was evaluated by therapists using the ground truth.

**Results:**

Over 98% of the LLM‐generated summaries aligned with the ground truth in terms of accuracy. Our evaluations showed an improved consistency in format and enhanced readability from the LLM‐generated summaries compared to the corresponding therapist‐generated summaries. This automated approach demonstrated consistent performance across all groups, regardless of treatment modality or disease site.

**Conclusions:**

This study demonstrated the high precision and consistency of the LLaMa 3.1 405B model in extracting keywords and summarizing CT simulation orders, suggesting that LLMs have great potential to assist in this task, reduce the workload of CT simulation therapists and improve radiation oncology workflow efficiency.

## 1 INTRODUCTION

In recent years, artificial intelligence (AI) has revolutionized a wide range of fields. Built on transformer architecture^1^, ^2^ and trained on a vast corpora of text data, large language models (LLMs) such as ChatGPT (OpenAI, San Francisco, CA)^3^ have demonstrated the ability to analyze complex text information,^4^, ^5^, ^6^ potentially assisting in decision‐making.^7^, ^8^, ^9^ LLMs have shown great capabilities to understand and summarize unstructured texts in healthcare.^10^, ^11^, ^12^, ^13^, ^14^ Meanwhile, commercially available LLMs have limitations, particularly in healthcare,^15^, ^16^, ^17^ where patient health information (PHI) protection is a major concern. The costs associated with large‐scale Application Programming Interface (API) service queries can also pose an additional financial charge to clinics. In radiation oncology, the complexity and precision required for cancer treatment procedures require extensive documentation.^18^, ^19^, ^20^, ^21^, ^22^, ^23^ Accurate records are essential for multidisciplinary coordination, patient safety, and positive outcomes.^24^ As the ongoing healthcare worker shortage and demand for healthcare services increase,^25^ efficient tools are needed to alleviate the documentation burden on healthcare professionals, allowing them to dedicate more time to patient care and other essential healthcare tasks. In radiation therapy, specifically CT simulation, CT simulation therapists manually summarize CT simulation orders prescribed by physicians into concise notes in the Epic electronic health record system (Epic Systems Corporation, Verona, WI). These therapist‐generated summaries are then transferred into the ARIA database (Varian Medical Systems, Palo Alto, CA) or another radiation oncology‐specific Electronic Medical Record (EMR) system on the patients’ CT simulation appointment page and serve as a general guidance for CT simulation therapists to set up the patient simulation. This process is tedious and can substantially increase the CT simulation therapists’ workload, especially with a high daily volume of patients or urgent cases. Moreover, this manual process of CT order summaries is prone to inconsistency, inefficient, and burdensome for therapists due to variation in writing styles. It also poses interpretation challenges for research teams.

To address these challenges, in this work, we investigated the use of LLMs to automate the summarization of CT simulation orders. The primary objective of this study is to evaluate the feasibility of an LLM‐assisted summarization tool to automatically generate CT simulation summaries in the radiation therapy setting. This work aimed to leverage LLM‐generated summaries to reduce variation in documentation, save time for therapists on repetitive tasks, improve workflow efficiency, and ultimately increase time allocated to direct patient care. Additionally, the study sought to explore the potential of such tools in alleviating clinical workloads during ongoing healthcare staff shortage. Instead of commercially available LLMs, our study used the locally hosted LLaMa 3.1 405B model (Meta, Menlo Park, CA)^26^ to automate the summarization of CT simulation orders. This approach protects patient privacy by keeping data local. Moreover, it addresses issues such as high costs, usage limits, and legal concerns, making internal hosting of LLaMa 3.1 405B a secure, reproducible, and compliant solution for our study.

## 2 MATERIALS AND METHODS

### 2.1 Data

**FIGURE 1:**
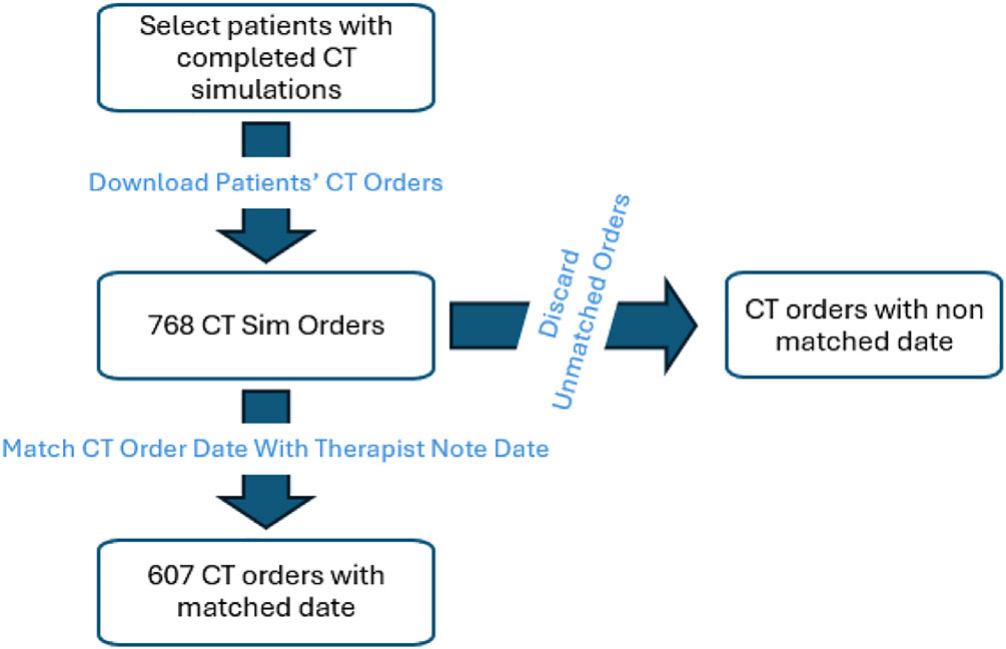
Pre‐processing the dataset by matching the exam dates. The raw dataset was processed by matching the exam date of the CT simulation orders with the date of the corresponding therapist‐written notes, retaining only matched CT simulation orders and discarding unmatched CT simulation orders. CT, Computed Tomography.

**FIGURE 2:**
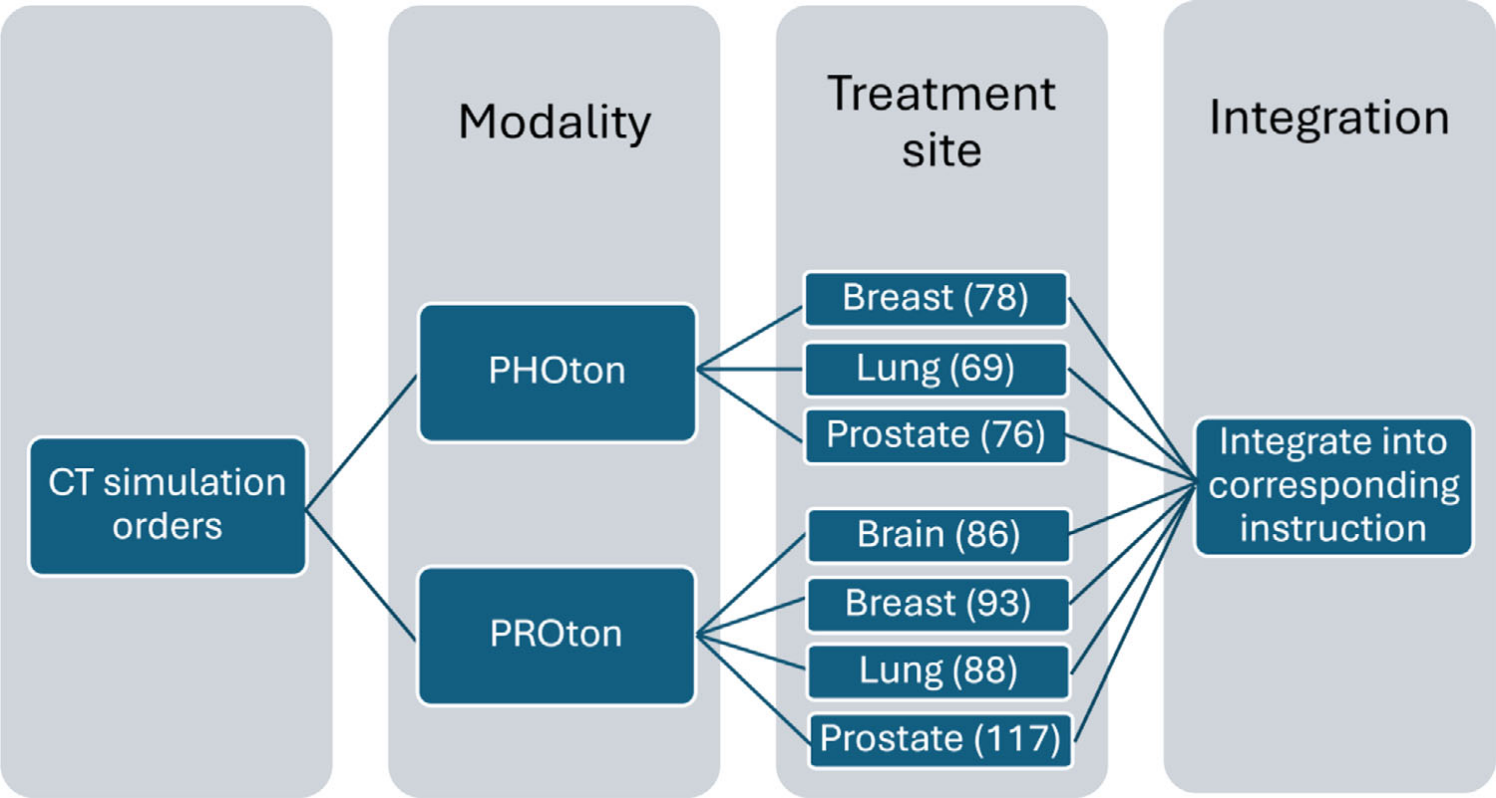
Categorization and integration of the dataset. The workflow demonstrates how data is systematically categorized by treatment modalities (such as proton or photon therapies) and disease sites, then data is categorized into 7 groups, ensuring data quality and consistency for analysis.

We utilized 768 patient cases whose CT simulations were completed after January 1st, 2019. The CT simulations were retrieved using SQL from the Aria database (ver.15.6) (Varian Medical Systems, Palo Alto, CA) through an in‐house patient data look‐up tool. 768 patients’ CT simulation orders and their corresponding therapist‐generated notes were downloaded. Orders were pre‐processed using Python to extract the treatment modalities and disease sites. From the 768 CT simulation orders, 7 groups categorized based on treatment modality and disease site were formed: proton (brain, breast, lung, prostate) and photon (breast, lung, prostate). Next, the CT simulation orders within these groups were matched with the therapist‐generated notes regarding their exam date, further narrowing down the number of samples to 607 CT simulation orders. To ensure data accuracy and clinical relevance, each CT simulation order was matched to the patient's most recent treatment record at the corresponding anatomical site. In this step, CT simulation orders associated with different treatment plans, anatomical sites, or rescheduled/canceled appointments were excluded. This final data set was used for further study, and the detailed selection process is presented in Figure 1. The number of CT simulation orders across each treatment category is presented in Figure 2.

### 2.2 LLaMa 3.1

This study primarily focused on the performance of the LLaMa 3.1 405B model and included a comparison with the 70B model for further analysis. The primary difference between the two models lies in their scale: the 70B and 405B variants contain approximately 70 billion and 405 billion trainable parameters, respectively. Although both models were trained on the same 15+ trillion‐token dataset,^27^ the 405B model demonstrates superior performance, achieving a higher MMLU benchmark score compared to the 70B model.^28^ Instead of utilizing the full‐precision LLaMa 3.1 405B or 70B models, we employed quantization^29^, ^30^ techniques to enable efficient inference on more modest hardware configurations, without significant compromise in performance. To enable the local deployment of the LLaMa 3.1 405B model within resource‐constrained clinical environments, model optimization techniques were employed to significantly reduce computational and memory demands. Specifically, 4‐bit weight quantization was applied, wherein high‐precision weights (typically 16‐ or 32‐bit floating‐point) were mapped to lower‐precision 4‐bit integer representations.^30^ This quantization reduced the model size by approximately 8‐fold, effectively decreasing GPU memory requirements and bandwidth consumption during inference. In parallel, FlashAttention,^31^ an optimized attention mechanism, was implemented to improve memory access efficiency and computation throughput. FlashAttention restructures traditional attention operations by fusing kernels and minimizing memory movement between computation and memory units.^32^ This approach improves runtime performance and Video Random Access Memory (VRAM) efficiency, particularly for long sequence inputs frequently encountered in clinical order summaries. These optimizations achieved a 75% reduction in hardware requirements, enabling the quantized model to be executed on 4 NVIDIA A100 80GB GPUs, in contrast to the 16 GPUs required for running the full‐precision version of LLaMa 3.1 405B. These techniques supported the cost‐effective and privacy‐preserving deployment of a high‐capacity LLM in clinical settings without reliance on cloud‐based services.

Moreover, we utilized the LLaMa 3.1 405B and 70B models through an internal API, ensuring strict patient privacy protection and adherence to relevant data security regulations. To assess the model's performance in summarizing CT simulation orders, we tested it at three different temperature settings: 0, 0.1, and 0.7, analyzing the impact on output consistency and accuracy. Additionally, the influence of model scale on summarization accuracy and reliability was evaluated via a comparison of Llama 3.1 405B and Llama 3.1 70B, with the 70B model tested at a fixed temperature of 0.1. To reduce the response variation, the temperature was set to 0.1 for LLaMa 3.1 405B. However, different temperature configurations (e.g., 0.7) were explored to check the impact of temperature upon the LLaMa 3.1 405B model's response.

In LLMs, the temperature parameter is a critical hyperparameter that modulates the randomness of token selection during text generation.^33^ The temperature is defined as below,

(1) $$Pi=\frac{e^{\frac{z_{i}}{T}}}{\sum_{j}e^{\frac{z_{j}}{T}}}$$

where i is the index of a specific token, and j is an index over all possible tokens. T is the temperature, and Pi and $z_{i}$ are the probability and model's logit assigned to token i, respectively. Lower temperature values (e.g., T approaching 0) make the model's output more consistent. It is important to note that while T = 0 is not mathematically defined in the Equation (1), it is treated as a special case in practice, where the model bypasses the computation and selects the most likely token at each step (i.e., greedy decoding).^34^, ^35^ Conversely, higher temperature settings introduce greater variability in the output.^36^, ^37^ In this study, we evaluated three different temperature settings using the LLaMa 3.1 405B model and selected the setting that yielded the best performance for subsequent comparisons with the LLaMa 3.1 70B model.

### 2.3 Prompt engineering

A prompt is the instruction given to LLM to guide it in producing desired outputs.^38^ Prompt engineering plays a critical role in guiding LLM to generate accurate CT simulation order summaries. In this study, the CT simulation therapists conducted a detailed review of the original CT simulation orders and developed standardized summary examples to support the initial prompt construction. Furthermore, with their assistance in clarifying the intent and meaning of key terms within the CT simulation orders, the prompts were refined using logically ordered bullet points. These refinements clearly outlined the rules for processing and output expectations, enabling the model to generate more reliable and accurate summaries across a wide range of clinical situations.

To account for different summarization requirements across different categories, a customized prompt was developed for each category. Generally, the prompt of each category will include these four parts: (1) set the role of the LLM, (2) provide the rules, (3) show examples, and (4) give guidance for the final output. First, the role of the LLM is defined as a professional medical assistant tasked with assisting the therapists in summarizing CT simulation orders. Second, the prompt provides a structured set of rules to guide the LLM in extracting the required information in a specific sequence and formatting it into standardized medical language. For example, for a CT simulation order specifying the proton modality to treat a lung cancer patient using deep‐inspiration breath hold (DIBH), the LLM would first identify the modality and format it as “PROton.” Then, it would determine the treatment site and append it with a space, resulting in “PROton Lung.” Details like motion management would be added after the treatment site, separated by a comma. In this example, the correct summary would be: “PROton Lung, DIBH.” Third, to improve the LLM's accuracy, a detailed example and its correct summary were included in the prompt. This serves as a reference to ensure consistency and accuracy in the output. Lastly, the guidelines specify the required output format. Given the tendency of the LLaMa 3.1 405B to explain processes, the rules of this step mandate that the output should concisely be in a JSON format, aligned with the example output. Additionally, the guidelines also emphasize that the LLM should generate the summary without including extraneous phrases or explanations. For example, the output should avoid starting with phrases like “Here is the summary” or describing the process.

**FIGURE 3:**
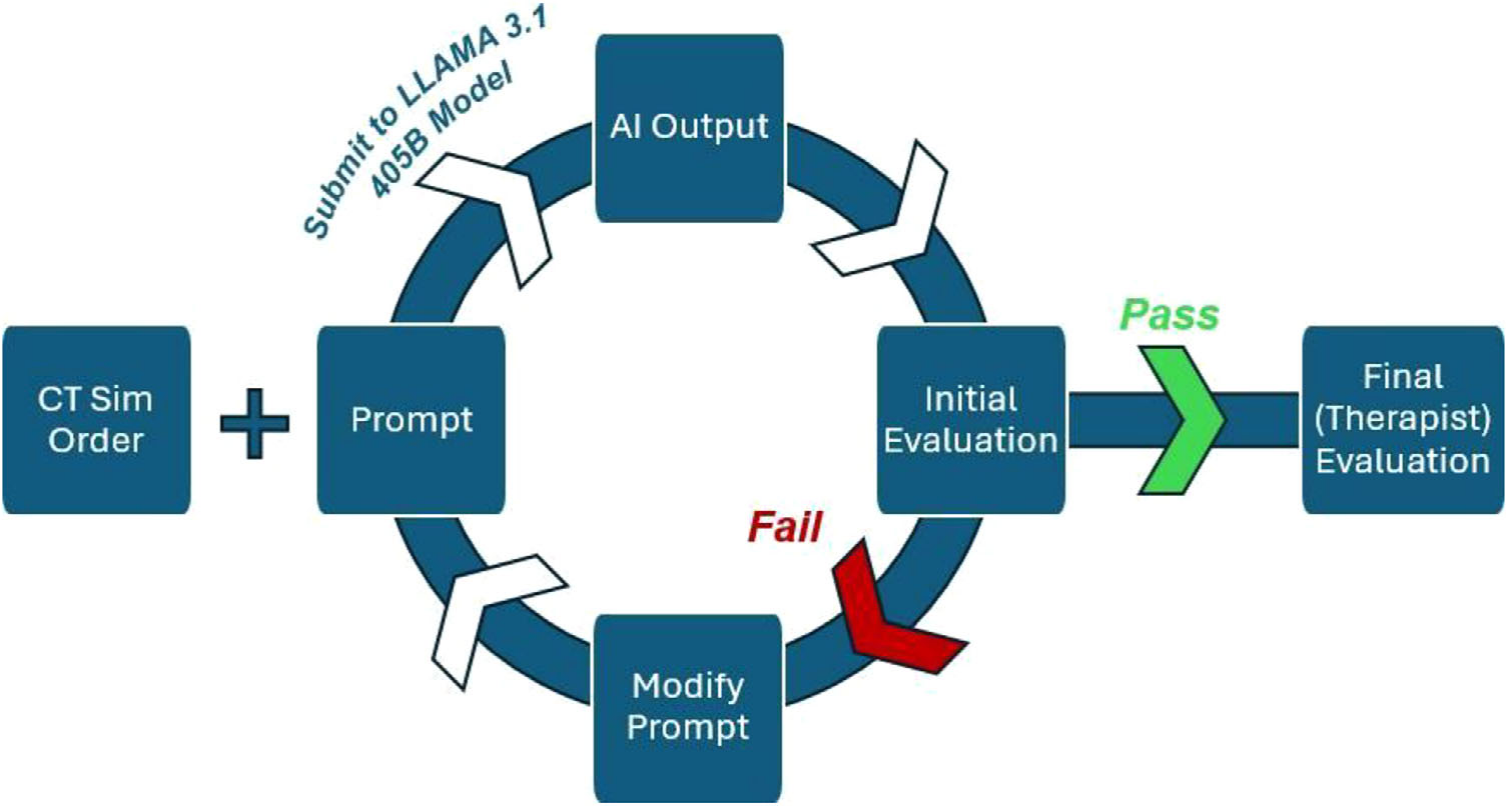
Prompt engineering and evaluation process. The LLM output generated from the customized prompt undergoes continuous evaluation until it meets the initial evaluation standards. During this process, the prompt is iteratively refined after each evaluation.

The prompts were not finalized at once. Prompts were iteratively refined based on the results generated by the model to increase accuracy and to account for the variations of the key information in the CT simulation order, as illustrated in Figure 3, including orders that deviated from the standard format. To enhance the model's ability to identify treatment sites, the rules in the prompts were added to include the information listed after “treatment site”, “treatment site 1/2,” or “anatomical sites.” Similar adjustments were made for other required fields to improve the precision and completeness of the CT simulation order summary. Details of the final prompt templates for the 7 categories can be found in the supplementary materials (Supplementary Materials, Section 5—Prompts).

To further evaluate response consistency, for each CT simulation order, the model was queried three times with the same prompt to further check its response consistency.

### 2.4 Evaluation

The performance of the LLM‐generated CT simulation order summaries was evaluated with two components: (1) Accuracy Evaluation Against Ground Truth; (2) Survey‐Based Therapists Evaluation of Consistency and Accuracy.

**Part 1: Accuracy evaluation against ground truth**

To establish a reference standard, experienced CT simulation therapists manually drafted standardized summaries for each CT simulation order, which served as the ground truth. The LLM‐generated summaries were then compared to these ground truth summaries using a word‐by‐word exact match criterion. Any LLM‐generated summaries that deviated from the ground truth were classified as a failed case.

**Part 2: Survey‐based evaluation of consistency and accuracy**

Six therapists self‐recorded the time spent manually drafting and reviewing summaries for 45 randomly assigned cases, and two therapists also self‐recorded the time spent reviewing an additional 21 cases with LLM‐generated summaries. Therapists were also asked to rate the consistency of nomenclature and the frequency of errors in both manual and LLM‐generated summaries using a 10‐point Likert scale. A score of 1 indicated that errors were found very frequently, while a score of 10 indicated that errors were never found. It is important to note that factors such as individual working styles, experience levels, subjective biases, and CT order categories were not considered in this survey and may have contributed to variability in the results.

## 3 RESULTS

The accuracy and consistency of the LLaMa 3.1 405B model‐generated results were reported. Accuracy represents the model's performance in adhering to the specified prompt rules, while consistency evaluates its reliability and stability across repeated evaluations under identical temperature settings. Table 1 presents an abbreviated Photon‐Prostate CT simulation order and its corresponding LLM‐generated summaries. A modified and de‐identified Photon‐Prostate CT simulation order was also provided in the Supplementary Materials (Section 6 – Sample CT Simulation Order) for demonstration. As shown in Table 2, despite similar CT simulation orders, therapists’ summaries show notable variability in style and phrasing, whereas the LLM‐generated summaries remain consistent with the ground truth. Moreover, the LLM‐generated summaries do not require additional drafting time from the therapists. Also, the clear and standard format makes it easier for the therapists to review and finalize, enabling them to focus more on patient care. Besides, as indicated in Table 1, although the discrepancies observed in laterality did not result in clinical errors, the therapists’ summaries not only show lower accuracy in noting the laterality of disease sites but also include redundant information.

**TABLE 1:** Abbreviated Photon‐Prostate CT Simulation Order with Corresponding LLM‐Generated Summaries.

Abbreviated CT simulation order	LLM generated CT order summary
MRN: 123456789	PHOton SBRT Prostate, Full Bladder, Rectal Ballon, MRI in Tx
Name: Test, Patient	
Gender: M	
Proton → No	
Treatment Site 1 → Prostate	
Laterality → None	
Special Instructions: → MRI in Tx position	
Bladder Options: → Full Bladder	
IV Contrast → No	
Contrast: → None	
Immobilization devices: → Rectal balloon	
Implanted medical device: → No	
Treatment Technique: → SBRT	

Abbreviations: CT, computed tomography; LLM, large language model.

**TABLE 2:** Comparison of the Therapists’ and LLM‐generated summaries for similar CT simulation orders.

Ground Truth	Therapists’ summaries	LLM‐generated summaries
“PROton Brain, Bolus helmet, MRI in Tx”	“PROTON PEDS ANES(5yo F) HELMET BRAIN, NEEDS SPANISH INTERPRETER; NO IV Cont; NO CHEMO, MR TX POSITION”	“PROton Brain, Bolus helmet, MRI in Tx”
	or	
	“PROton Brain PEDS/ SKA / BOLUS HELMET, NO Ans/ RN: 10 / MR:1:15″	
	or	
	“PROton BRAIN. helmet. RN: 130. MR in tx pos”	
“PHOton SBRT Lt Lung, 4DCT, IV contrast”	“PHOTON SBRT LT LUNG, 4D, IV CST”	“PHOton SBRT Lt Lung, 4DCT, IV contrast”
	Or	
	“Photon SBRT Lung, IV Contrast, 4D”	
“PHOton Prostate—Bed, Full Bladder With and Without Rectal Balloon, MRI in Tx”	“PHOTON PROSTATE BED, SCAN W/ AND W/O RECTAL BALLOON”	“PHOton Prostate—Bed, Full Bladder With and Without Rectal Balloon, MRI in Tx”
	or	
	“PHOTON PROSTATE BED, NO RB”	
“PHoton Lt Breast, DIBH”	“PHoton Lt Breast, DIBH”	“PHoton Lt Breast, DIBH”
	Or	
	“PHOTON LT BREAST DIBH”	

Abbreviations: CT, computed tomography; LLM, large language model.

Therapists’ assessments of nomenclature consistency and accuracy are summarized in Table 3. On average, the manual process, which involved both creating and reviewing CT simulation order summaries, took about 6.1 min per case. Whereas the LLM‐assisted process, which required only reviewing the AI‐generated summaries, took less than 1 min per case. This reduction is likely due to both the higher accuracy and the more standardized format of the LLM‐generated summaries, which minimized the need for therapists to make modifications during their review.

**TABLE 3:** Quantitative evaluation of manual and LLM‐assisted CT simulation order summary generation.

	Manual process	LLM‐assisted process
Average time per CT simulation order summary	6.1 ± 2.1 min	0.7 ± 0.2 min
Average accuracy satisfaction score	6.8/10	8.0/10
Average consistency satisfaction score	6/10	9.0/10

Abbreviations: CT, computed tomography; LLM, large language model.

**FIGURE 4:**
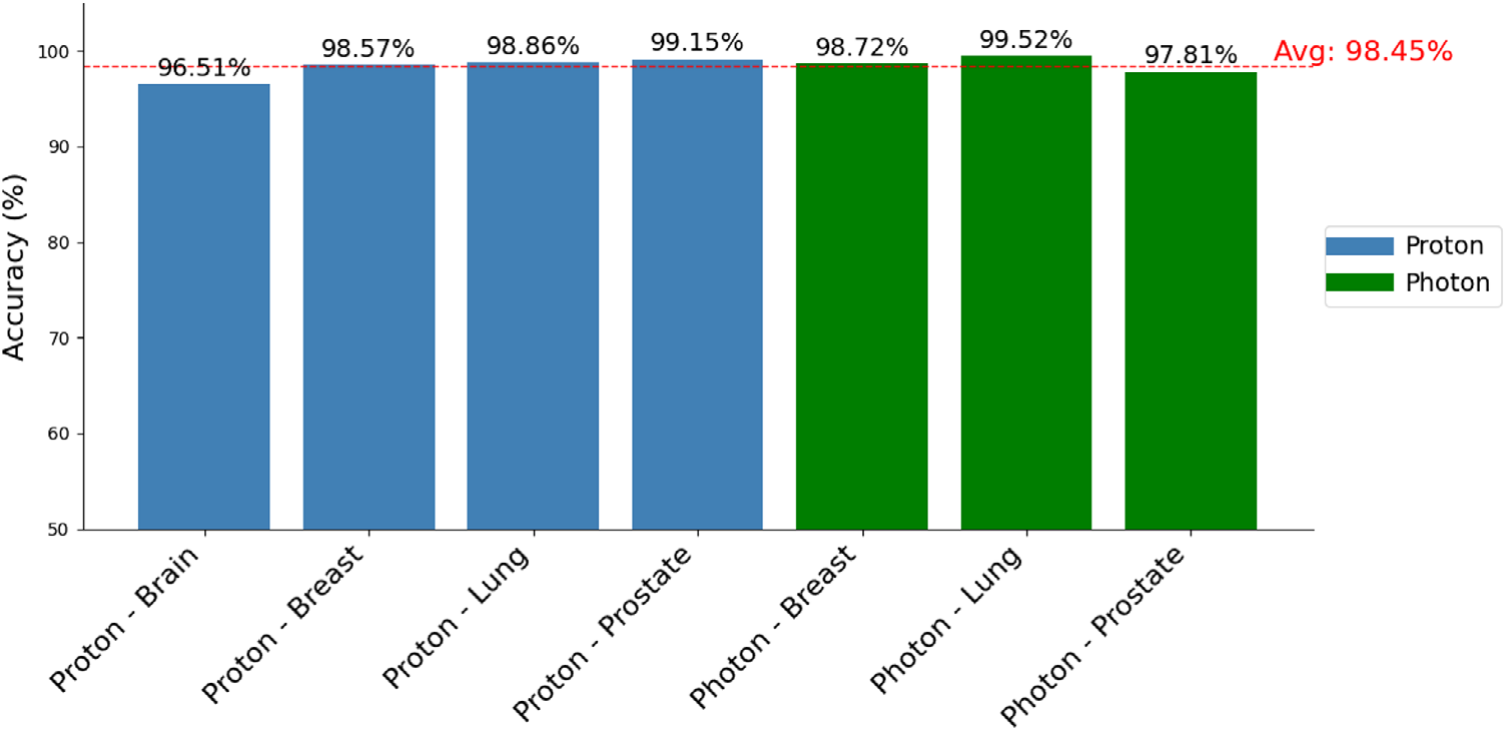
The accuracy of the LLaMa 3.1 405B (*T* = 0.1) generated summaries across 7 groups. Each color in the circle represents a specific category, with the corresponding accuracy of the LLM‐generated summaries for that group shown in the same color. LLM, large language model.

As shown in Figure 4, in the expert evaluation by the therapist, the LLM‐generated summaries had an average accuracy of 98.45% among all seven categories, with the photon‐lung group having the highest accuracy (99.5%). The lowest accuracies were observed for the photon‐prostate (97.8%) and proton‐brain (96.5%) groups.

Moreover, consistently high accuracies were observed for the LLaMa 3.1 405B model using different temperature settings. The temperature configuration of 0.1 yielded the highest accuracy in summarizing CT simulation orders. Among three trials of the same prompt, the LLM‐generated summaries also showed great consistency in accuracy (about 98%). Details of the accuracies obtained at different temperatures, along with the consistency results from three trials, were provided in the supplementary material (Supplementary Materials, Section 2—Accuracy). At a temperature configuration of 0.1, LLaMA‐3.1‐70B achieved approximately 90% mean accuracy on the LLM‐generated summaries, underperforming the 405B model. Similarly, details of the LLaMa 3.1 70B results on this task were included in section 2 of the supplementary material.

## 4 DISCUSSION

### 4.1 Benefits of LLM‐generated CT simulation order summaries

Manually generated CT simulation order summaries are often time‐consuming and prone to inconsistency and error. The integration of LLM‐generated summaries into the CT simulation workflow offers measurable improvements in efficiency, accuracy, and standardization. By automating the drafting process, LLM‐generated summaries do not take the therapists’ time to draft the summaries and can reduce the time by more than 80% per case. This substantial reduction in workload can significantly alleviate documentation burdens in high‐volume clinical settings.

Additionally, survey data from Table 3 also revealed only moderate satisfaction with the consistency (mean score: 6.0/10) and accuracy (mean score: 6.8/10) for the manually drafted summaries, and LLM‐generated summaries outperformed the manual ones in both evaluations. Although the therapist ratings were lower than the measured accuracy for LLM‐generated summaries, this discrepancy is largely attributable to differences in work experience and a lack of standardization in the CT order summaries within current workflow. Furthermore, all participating therapists expressed strong support for the adoption of standardized nomenclature. Together, these findings highlight the urgent need for a standardized, well‐structured, accurate, and time‐saving solution in the current CT simulation order summary generation workflow. In this context, the clear and structured nature of LLM‐generated summaries not only enhances interdisciplinary communication among therapists but also enhances communication across different teams within the department, including physicians, physicists, dosimetrists, nurses, and researchers. LLM‐generated summaries can also serve as practical reference examples for newer or less experienced team members. Meanwhile, although patient‐specific cases require careful customization, automating CT simulation order summaries with LLMs can be particularly beneficial in standardized clinical settings where protocols and treatment setups are consistent, enabling the model to generate highly accurate and reliable summaries due to reduced ambiguity and variation in patient‐specific comments.

### 4.2 Patient data processing

In this study, 607 CT simulation orders were included in the final analysis. This number differed from the initially selected orders retrieved from the internal data system due to the patient identification number, disease site, and exam date selection criteria. The CT simulation order selection process was essential for eliminating duplicates, rescheduled CT simulation orders, and orders associated with undesired disease sites. Multiple disease sites for the same patient, rescheduling CT simulations, or other unusual scenarios in patients’ CT simulations can result in multiple CT simulation orders. Deterministic linkage on patient identification number, disease site, and CT simulation date was used to confirm concordance between physician CT simulation orders and therapist notes for the same encounter. Subsequently, verified records were grouped by treatment modality and disease site.

### 4.3 Review of the LLM generated summaries

As shown in Figure 4, the largest accuracy difference was observed between the Proton‐Brain category (96.51%) and the Proton‐Prostate group (99.15%). This discrepancy may be partially attributed to the variation in sample sizes between the two groups. Specifically, the Proton‐Prostate group included 117 CT simulation orders, whereas the Proton‐Brain group contained only 86. With two cases in the Proton‐Prostate group and three in the Proton‐Brain group where the summaries generated did not fully align with the ground truth, this difference in accuracy highlights the sensitivity of the evaluation metric to small variations in performance when sample sizes are limited (see Section 4.4 for details). The accuracy of the LLM‐generated summaries was assessed using a strict word‐by‐word comparison with the ground truth, where only exact textual matches were counted as correct. While reviewing the summaries generated by the LLaMa 3.1 405B model, subtle differences were observed for a few cases, such as missing the disease site mentioned in the comments or inaccuracies in key information. These discrepancies were primarily attributed to ambiguities in the CT simulation orders. For example, for PROton‐brain patients’ CT simulation orders, instructions regarding bolus can be confusing to the LLM model. Normally, the CT simulation order may present the bolus information under the bolus section. However, this information can appear inconsistently, such as “Bolus → Yes; Bolus → No” or “Bolus → Yes; comment → without bolus,” or it may be detailed only in the physician's note section. In this case, the therapists were instructed to perform the CT simulation first with a bolus and then repeat the process without a bolus. However, for the LLM to interpret these instructions correctly and generate an appropriate summary, additional contextual knowledge within the CT simulation orders is necessary. This variability in CT simulation orders introduced challenges in the bolus‐related sections of the summaries, affecting the model's accuracy and consistency. Interestingly, while the 405B models occasionally generate inaccurate summaries for CT simulation orders, they produce correct summaries when the same orders are processed via the internal web portal. This inconsistency likely arises from the models’ sensitivity to handling large datasets concurrently or inherent limitations in managing complex inputs, despite receiving structured prompts in a similar format.^39^, ^40^

In real‐world clinical settings, the final CT simulation could end up quite differently from the original physician's CT simulation order, which can be due to patient‐specific factors identified before or during simulation, resulting in simulation cancellation or modification. For example, simulations may be canceled if a patient with mycosis fungoides shows improvement with topical therapy, or expanded if new symptoms arise, such as lower back pain in a patient initially referred to as breast simulation. In these scenarios, the CT simulation therapists will prepare new documents noting patients’ setup details. However, the goal of this study is to automate the generation of the CT simulation summaries based on the physicians’ CT simulation orders to streamline the preparation of the patient CT simulation and reduce the workload of the CT simulation therapists by minimizing the redundant and repetitive clinical tasks. Since major changes are uncommon within the short time between physicians’ CT simulation orders and patient CT simulations, the LLM‐generated summaries remain a reliable tool for guiding the initial patient setup for CT simulation under therapist oversight.

### 4.4 Analysis of failed cases

It is important to note that a failed case in this context does not necessarily indicate a clinically misleading summary. The most common types of discrepancies observed were related to keyword mismatches and inconsistent phrasing, for example, including unneeded details (such as “no rectal balloon”) or synonymous phrasings (such as “breast bolus” and “breast immobilization”). These discrepancies often arose from variability or ambiguity in the physician's CT simulation orders, resulting in reduced accuracy of the LLM‐generated summaries.

The performance of this model can be improved by further refinement of the prompts. During prompt engineering, accuracy was notably enhanced when prompts explicitly listed all possible scenarios for identifying keywords. On the other hand, recent research suggests that the structures and specifications of the prompts can significantly influence the logical coherence and output quality, as the way in which the prompt is framed shapes the model's internal reasoning pathway.^41^ Therefore, for prompt refining, in addition to enumerating all possible scenarios, different prompt structures should be tested to identify which prompt format yields optimal results. Meanwhile, it is crucial to have post‐processing processes to manage variability and conflicting contexts in the CT simulation orders, such as flagging inconsistencies. Lastly, therapists’ oversight remains essential, especially for some complex cases that require clarification from the physicians. By applying these strategies, failed cases can be minimized, enabling the LLM‐generated summaries to be reliable despite the variations in CT simulation orders.

### 4.5 Complexity of the clinical data

Although the overall accuracy of the LLaMa 3.1 405B model exceeds 98%, PHOton‐ Breast, PHOton/PROton‐Prostate, and Proton‐Brain groups exhibit lower accuracy when compared to other groups. This accuracy variation among categories reflects the complexity and variability of the clinical data within each group. For example, within the same group of CT simulation orders, certain critical details may vary from one to another. When bolus helmet information is present in the PROton‐Brain CT simulation orders, it may refer to the mask for the CT simulation or the actual bolus helmet used during the radiation therapy. Thus, the actual use of a bolus helmet will need to be confirmed based on the patient's clinical notes.

Moreover, the amount of required information in the summarized notes differs across treatment modalities. For example, the PHOton Breast group requires six key pieces of information: Treatment Modality, Disease Sites, Laterality, IV Contrast, Motion Management, and Implanted Medical Device. In contrast, the PHOton Prostate group demands ten pieces of information: Treatment Modality, Disease Sites, Special Instructions, MRI in Treatment Position, Bladder Options, IV Contrast, Treatment Techniques, Chemo Coordination, Motion Management, and Implanted Medical Device. These variations in complexity and specificity of required information could pose a challenge to the LLMs and result in varied accuracies across different groups, as shown in Figure 4.

Lastly, the complexity and internal consistency of the CT simulation orders can significantly impact the accuracy of the LLM‐generated summaries, especially in cases where conflicting or ambiguous information is present. For example, a general note/comments field in the CT simulation order indicates a disease in the left breast, while the laterality field in the structured CT simulation order specifies the right side. The model may produce an incorrect output since the model prioritizes the structured CT simulation order. In this case, it is crucial to enhance the model's ability to flag inconsistencies and to maintain the oversight by CT simulation therapists to validate and rectify the outputs when needed.

### 4.6 Quantized LLMs deployment in various healthcare systems

Despite being a general‐purpose LLM without task‐specific fine‐tuning, the LLaMa 3.1‐based system demonstrated a strong capacity to generalize across diverse phrasing and content structures within CT simulation orders. Most discrepancies that we observed were not due to misunderstanding clinical contexts but over‐interpretation or documentation mismatches. These cases underscore the model's sensitivity to contextual cues and the importance of prompt optimization in clinical applications.

One of the key challenges in integrating LLM into clinical workflows lies in the inherent variability of documentation practices across different health systems. CT simulation orders often exhibit non‐standardized language, physician‐specific notes, and inconsistent formatting that can introduce ambiguity and reduce interoperability. Nevertheless, the quantized LLaMa 3.1 models in this study can help address these challenges in a scalable and accessible way and serve as a flexible foundation for adapting LLMs to the needs of complicated and variable clinical environments. More importantly, it allows institutions to iteratively refine the model through prompt engineering, error analysis, and, if needed, lightweight fine‐tuning based on their own clinical documentation styles.

### 4.7 Limitation of current study

The results of this study are based on limited treatment categories and may not fully reflect the full diversity of clinical scenarios encountered in CT simulation. Also, though the LLaMa 3.1 405B model performed well in most cases, it remained sensitive to variation and inconsistency within the simulation orders. Even when all potential keyword scenarios were explicitly enumerated, the model occasionally produced inconsistent summaries across different trials for complex cases. Therefore, future work will focus on expanding the data set and refining the prompts and exploring fine‐tuning strategies to improve the model's robustness if needed.

## 5 CONCLUSION

Our study demonstrates the high accuracy and versatility of using an LLM in generating summaries for CT simulation orders. Our findings showed that quantized LLMs can be potentially integrated into the CT simulation workflow in radiation oncology, enhancing consistency, improving efficiency, and reducing the workload associated with CT simulation for therapists.

## AUTHOR CONTRIBUTIONS

**Meiyun Cao** and **Shaw Hu** contributed equally to this work and share first authorship. Both were responsible for the conceptualization of the study, data curation, analysis, and initial drafting of the manuscript. **Jason Sharp** provided essential input in prompt setup, verified results, and contributed to manuscript proofreading and clinical validation. **Edward Lee Clouser** assisted with data processing and participated in protocol discussions to refine study procedures. **Jason M. Holmes** assisted with data processing and participated in protocol discussions to refine study procedures. **Linda L Lam** aided in data handling and contributed ideas during protocol formulation and team discussions. **Xiaoning Ding** participated in prompt evaluation, shared feedback on analysis strategy, and assisted with figures and tables. **Diego Santo Toesca** assisted with data processing and participated in protocol discussions to refine study procedures. **Brianne C Raulston** assisted with data processing and participated in protocol discussions to refine study procedures. **Wendy S Lindholm** assisted with data processing and participated in protocol discussions to refine study procedures. **Samir H. Patel** provided support in discussions around methodological improvements and contributed comments during manuscript editing. **Sujay A Vora** provided support in discussions around methodological improvements and contributed comments during manuscript editing. **Peilong Wang** and **Wei Liu** served as corresponding authors, provided senior‐level guidance on study design, critically reviewed the results, revised the manuscript, and oversaw the overall direction and integrity of the project. All authors reviewed and approved the final manuscript.

## CONFLICT OF INTEREST STATEMENT

Meiyun Cao, Shaw Hu, Jason Sharp, Edward Lee Clouser, Jason M. Holmes, Linda L Lam, Xiaoning Ding, Diego Santos Toesca, Brianne C Raulston, Wendy S Lindholm, Sujay A Vora, and Peilong Wang declare no relevant conflicts of interest to disclose. Samir H. Patel discloses that he received an honorarium from Galera Therapeutics for providing feedback on commercialization efforts. Wei Liu declares his royalties at the Mayo Clinic and holds multiple patents, disclosures, and copyrighted software, some of which are licensed to Decimal LLC.

## Supporting information



Supporting Information
