# Supplementary material for: Using large language models to automate summarization of CT simulation orders in radiation oncology
Source: J Appl Clin Med Phys. 2025 Oct 27;26(11):e70310. doi: 10.1002/acm2.70310 (PMC12558592; doi:10.1002/acm2.70310)
Supplement: Supplementary file 1 — Supporting Information [file ACM2-26-e70310-s001.pdf]

Supplementary Materials

1 Introduction to supplementary materials

This supplementary material provides a detailed comparison of accuracy, repeatability, and similarity between different LLAMA models under various temperature settings. In addition, it includes all the seven prompts used in the study.

2 Accuracy

Table 1 illustrates the accuracy of 3 trials among the LLAMA 3.1 405B(T = 0, 0.1, 0.7) and 70B (T = 0.1) models. The results show that the 70B model is less effective in adhering to the established formatting rules outlined in the prompt compared to the 405B model, which resulted in missing, additional, or incorrect information in the generated summaries. The average accuracies for LLAMA 3.1 405B(T = 0) and LLAMA 3.1 405B(T = 0.7) are 97. 86% and 98. 16%, respectively, which are slightly lower than LLAMA 3.1 405B(T = 0.1). Table 2 presents a specific number of inaccurate summaries for each category of LLAMA 3.1 405B (T = 0.1).

Table 1: Accuracy for all models

**Accuracy**	**405B T0**	**405B T0.1**	**405B T0.7**	**70B T0.1**
Trial 1	97.40%	98.98%	98.46%	90.00%
Trial 2	98.08%	98.11%	97.96%	90.16%
Trial 3	98.11%	98.25%	98.07%	90.75%
Average accuracy	97.86%	98.45%	98.16%	90.47%

Table 2: Accuracy for 3 trails for each category

	Inaccurate AI summaries recorded		
**Accuracy**	**Trail 1**	**Trial 2**	**Trial 3**
PHO -Breast	1	1	1
PHO-Lung	0	0	1
PHO-Prostate	1	2	2
PRO-Brain	2	3	4
PRO-Breast	1	2	1
PRO-Lung	2	1	1
PRO-Prostate	0	3	3

3 Repeatability

Repeatability was evaluated by exact matches of AI-generated summaries across three trials. Variations in formatting, capitalization of words, or the addition or omission of extra letters or symbols resulted in mismatched outputs, thereby lowering repeatability. Table 3 compares the repeatability and overall accuracy of the LLAMA 3.1 405B and 70B models across different temperature settings. In this table, the 405B model consistently demonstrates high repeatability across all temperatures, with values exceeding 92%, whereas the 70B model exhibits significantly lower repeatability at 74.01%. This suggests that the 405b model is more stable in generating outputs across repeated trials. For the 405B model, although an increase in repeatability was expected with a decreased temperature setting, though slightly. The repeatability remained the same at T = 0.1 and T = 0.7, with a slightly higher value observed at T = 0. A similar trend was observed in Table 1, where the 405B model at T = 0.1 achieved the highest accuracy in both metrics. However, the 70B model demonstrates a significantly lower repeatability of 74.01% at T=0.1. This discrepancy is probably attributed to the 405B model's superior ability to adhere to the rules specified in the prompt than the 70B model.

Table 3: Compare the 405B and 70B at temperature = 0.1

	**405b T0**	**405b T0.1**	**405b T0.7**	**70b T0.1**
repeatability(trial 1 vs trial 2)	93.44%	92.34%	92.03%	72.81%
repeatability(trial 1 vs trial 3)	94.22%	92.97%	92.97%	75.47%
repeatability(trial 3 vs trial 2)	95.16%	92.81%	92.81%	73.75%
Average repeatability	92.27%	92.60%	92.60%	74.01%

4 Similarity

The similarity metric is developed by cross comparing two corresponding individual AI generated summaries word by word. A failed similarity match includes mismatched the capitalization, missing or extra information. Table 4 presents the similarity metrics across model configurations. The average similarity between the 405B configurations is above

92%, with the highest being 93.33% between T=0.1 and T=0. The similarities among the 3 temperatures with the 405B model are less than 1%. However, the similarity between the 405B and 70B models (T=0.1) drops significantly to 72.22%, highlighting a notable difference in output consistency and quality between the two models.

Table 4: Similarity Across All Models and Temperatures

**Similarity**	**405B T0 vs 405B T0.1**	**405B T0.7 vs 405B T0.1**	**70B T0.1 vs 405B T0.1**	**405B T0 vs 405B T0.7**
Trial 1 vs Trial 1	92.97%	92.19%	72.34%	92.19%
Trial 1 vs Trial 2	94.38%	92.97%	70.31%	92.03%
Trial 1 vs Trial 3	92.34%	94.22%	72.50%	93.59%
Trial 2 vs Trial 1	94.69%	92.50%	72.62%	94.22%
Trial 2 vs Trial 2	91.97%	92.97%	70.62%	93.75%
Trial 2 vs Trial 3	93.28%	93.12%	73.44%	95.16%
Trial 3 vs Trial 1	93.12%	92.19%	73.44%	92.18%
Trial 3 vs Trial 2	94.22%	92.50%	71.25%	92.50%
Trial 3 vs Trial 3	92.97%	91.88%	73.44%	93.59%
Average Similarity	93.33%	92.73%	72.22%	93.32%

5 Prompts

Seven customized prompts are shown below:

5.1 PHOton - lung

```
You are a professional medical assistant that generate CT simulation
   order summary with a JSON object containing the relevant
   information: "modality, treatment technique, laterality, treatment
   site(s), motion managenment/sim technique, IV contrast, chemo
   condition, and implanted medical devices."
Here is the instruction of how to summarize each catogory:
For modality, if 'Proton->Yes', write down PROton, if 'Proton->No',
   write down PHOton.
For treatment technique, you will only need to write down treatment
   technique if it is "Electron", "SRS", "SRT", or "SBRT". Otherwise,
   leave it empty.
For Laterality, if Laterality->Right, abbreviate it as Rt in this part
   . If Laterality->Left, abbreviate it as Lt.
For treatment sites, find all treatment sites.
For Motion Management or Sim technique, only record this part when
   found DIBH and/or 4DCT. Otherwise, leave it empty.
For IV contrast, if 'IV Contrast -> Yes', write down 'IV contrast'.
   Otherwise, if 'IV Contrast -> No', or not found this infomation,
   leave it empty.
For Chemo condition, if 'Chemo Coordination->Yes', write down 'Chemo'.
    Otherwise, if 'Chemo Coordination-> No', or not found this
   infomation, leave it empty.
For Implanted Medical Device, if 'Implanted Medical Device->Yes',
   record 'Implanted Medical Device' in the summary. Otherwise, leave
   it empty.
Here is an example CT simulation order and the correct summary with a
   JSON object:
''''
Example CT simulation order:
"
Proton->NO
Treatment Technique:->SBRT
Laterality->Right
Treatment Site->Lung
Motion Management -> None
IV Contrast->Yes
Implanted medical device:->Yes
Chemo conditon -> None
"
Here is the coresponding correct CT order summary with a JSON object:
   {"modality, treatment technique, laterility and site(s)": "PHOton
   SBRT Rt Lung", "Motion Management or Sim technique": "", "IV
   contrast": "IV contrast", "Chemo condition":"", "Implanted medical
   device":"Implanted medical device"}. If the part is not found,
   please leave it empty. Do not add extraneous data to explain the
   process after the closing of this Jason format. Do not add comma
   after the last key value pair.
''''
Now, using these rules, create a order summary with a JSON object as
   show in the example without explain the process.
```

```
Here is the physician order for patient simulation:

'''
[Insert Physician Order In Here]

'''
Please do not start with: "Here is the physician report summary:".
```

5.2 PHOton - prostate

```
You are a professional medical assistant that generate CT simulation
   order summary with a JSON object containing the relevant
   information: "modality, treatment technique, treatment site(s),
   Bladder Options and Immobilization devices, IV contrast, chemo
   condition, bolus condition, MRI in Tx condition,  implanted medical
    devices".

Here is the instruction of how to summarize each catogory:
For modality, if 'Proton->Yes', write down PROton, if 'Proton->No',
   write down PHOton.
For treatment technique, you will only need to write down treatment
   technique if it is "Electron", "SRS", "SRT", or "SBRT". However, if
    the Treatment Technique corresponds to "Proton", "IMRT", "3D
   conformal", or "2D", leave it empty. Example:If Treatment Technique
    is 'SBRT', write 'SBRT'.If Treatment Technique is 'IMRT', write
   nothing (leave the field empty).
For treatment sites, find all treatment sites.
For Bladder Options and Immobilization devices, only record this part
   when found full/partial/empty bladder, and/or Rectal Balloon.
   Otherwise, leave it empty.
For IV contrast, if 'IV Contrast -> Yes', write down 'IV contrast'.
   Otherwise, if 'IV Contrast -> No', or not found this infomation,
   leave it empty.
For Chemo condition, if 'Chemo Coordination->Yes', write down 'Chemo'.
    Otherwise, if 'Chemo Coordination-> No', or not found this
   infomation, leave it empty.
For Bolus, if 'Bolus' contains a value other than 'None', summarize
   the type of bolus and record it in the summary. If 'Bolus:->Other,
   see comments', use the comment to determine the type of bolus.
For MRI condition, only check if "MRI in Tx" and/or "TP MRI", record '
   MRI in Tx' for the MRI in Tx condition section. Otherwise, leave it
    empty.
For Implanted Medical Device, if 'Implanted Medical Device->Yes',
   record 'Implanted Medical Device' in the summary. Otherwise, leave
   it empty.


Here is an example CT simulation order and the correct summary with a
   JSON object:
''''
Example CT simulation order:
"
Proton->NO
Treatment Site->Prostate
Motion Management -> Full Bladder
IV contrast -> No
Immobolization devices: ->  Rectal Balloon
Immobilization comment:->Prostate Leg Immobilizer
```

```
Special Instructions:-> MRI in Tx position
Treatment Technique:->SBRT
Fusion Comment-> MRI
Implanted Medical Device->Yes
"
Here is the coresponding correct CT order summary with a JSON object:
   {"modality, treatment technique, and site(s)": "PHOton SBRT
   Prostate", "Bladder Options and Immobilization devices": "Full
   Bladder, Rectal Balloon", "IV contrast": "", "Chemo Coordination
   ":"", "MRI in Tx condition": "MRI in Tx", "Implanted Medical Device
   ": "Implanted Medical Devices"}.If the part is not found, please
   leave it empty. Do not add extraneous data to explain the process
   after the closing of this Jason format. Do not add comma after the
   last key value pair and make sure the Json format is accurate.
''''
Now, using these rules, create a order summary with a JSON object as
   show in the example without explain the process.

Here is the physician order for patient simulation:

'''
[Insert Physician Order In Here]

'''
Please do not start with: "Here is the physician report summary:".
```

5.3 PHOton - breast

```
You are a professional medical assistant that generate CT simulation
   order summary with a JSON object containing the relevant
   information: "modality, treatment technique, laterality, treatment
   site(s), motion managenment/sim technique, IV contrast, chemo
   condition, bolus, and implanted medical devices."
Here is the instruction of how to summarize each catogory:
For modality, if 'Proton->Yes', write down PROton, if 'Proton->No',
   write down PHOton.
For treatment technique, you will only need to write down treatment
   technique if it is "Electron", "SRS", "SRT", or "SBRT". Otherwise,
   leave it empty.
For Laterality, if Laterality->Right, abbreviate it as Rt in this part
   . If Laterality->Left, abbreviate it as Lt.
For treatment sites, summarize all treatment sites and anatomical
   sites.
For Motion Management and/or Sim technique, only record this part when
    found DIBH and/or 4DCT. Otherwise, leave it empty.
For IV contrast, if 'IV Contrast -> Yes', write down 'IV contrast'.
   Otherwise, if 'IV Contrast -> No', or not found this infomation,
   leave it empty.
For Chemo condition, if 'Chemo Coordination->Yes', write down 'Chemo'.
    Otherwise, if 'Chemo Coordination-> No', or not found this
   infomation, leave it empty.
For Bolus, if 'Bolus' contains a value other than 'None', summarize
   the type of bolus and record it in the summary. If 'Bolus:->Other,
   see comments', use the comment to determine the type of bolus.
For Implanted Medical Device, if 'Implanted Medical Device->Yes',
   record 'Implanted Medical Device' in the summary. Otherwise, leave
   it empty.
```

```
Here is an example CT simulation order and the correct summary with a
   JSON object:
''''
Example CT simulation order:
"
Proton->No

Treatment Site 1->Breast

Laterality->Left

IV Contrast->No

Sim Technique:->CT only

Sim Technique:->DIBH

Bolus:->None

Immobilization devices:->Mask

Mask options:->Other, see comments

Immobilization comment:->Thorax Mask

Implanted medical device:->Yes

Implanted medical device comment:->sacral nerve stimulator will be
   placed after sim but before tx

Treatment Technique:->Proton single Iso

"
Here is the coresponding correct CT order summary with a JSON object:
   {"modality,treatment technique, laterility and site(s)": "PHOton Lt
    Breast", "Motion Management or Sim technique": "DIBH", "IV
   contrast": "", "Chemo condition":"Chemo", "Bolus": "", "Implanted
   Medical Device": "Implanted Medical Device"}. If the part is not
   found, please leave it empty. Do not add extraneous data to explain
    the process after the closing of this Jason format. Do not add
   comma after the last key value pair.
''''

Now, using these rules, create a order summary with a JSON object as
   show in the example without explain the process.

Here is the CT simulation order:

'''
[Insert Physician Order In Here]

'''
Please do not start with: "Here is the CT simulation order summary:".
```

5.4 PROton - lung

```
You are a professional medical assistant that generate CT simulation
   order summary with a JSON object containing the relevant
```

```
   information: "modality, treatment technique, laterality, treatment
   site(s), motion managenment/sim technique, IV contrast, chemo
   condition, and implanted medical devices."
Here is the instruction of how to summarize each catogory:
For modality, if 'Proton->Yes', write down PROton, if 'Proton->No',
   write down PHOton.
For treatment technique, you will only need to write down treatment
   technique if it is "Electron", "SRS", "SRT", or "SBRT". Otherwise,
   leave it empty.
For Laterality, if Laterality->Right, abbreviate it as Rt in this part
   . If Laterality->Left, abbreviate it as Lt.
For treatment sites, find all treatment sites.
For Motion Management or Sim technique, only record this part when
   found DIBH and/or 4DCT. Otherwise, leave it empty.
For IV contrast, if 'IV Contrast -> Yes', write down 'IV contrast'.
   Otherwise, if 'IV Contrast -> No', or not found this infomation,
   leave it empty.
For Chemo condition, if 'Chemo Coordination->Yes', write down 'Chemo'.
    Otherwise, if 'Chemo Coordination-> No', or not found this
   infomation, leave it empty.
For Implanted Medical Device, if 'Implanted Medical Device->Yes',
   record 'Implanted Medical Device' in the summary. Otherwise, leave
   it empty.

Here is an example CT simulation order and the correct summary with a
   JSON object:
''''
Example CT simulation order:
"
Proton->Yes
Treatment Technique:->SBRT
Laterality->Right
Treatment Site->Lung
Motion Management -> 4DCT
Sim techniques -> DIBH
IV Contrast->Yes
Chemo conditon -> Yes
Implanted Medical Device-> No
"
Here is the coresponding correct CT order summary with a JSON object:
   {"modality, treatment technique, laterility and site(s)": "PROton
   SBRT Rt Lung", "Motion Management or Sim technique": "4DCT DIBH", "
   IV contrast":"IV contrast", "Chemo condition":"Chemo", "Implanted
   Medical Device": ""}. If the part is not found, please leave it
   empty. Do not add extraneous data to explain the process after the
   closing of this Jason format. Do not add comma after the last key
   value pair.
''''
Now, using these rules, create a order summary with a JSON object as
   show in the example without explain the process.

Here is the physician order for patient simulation:

'''
[Insert Physician Order In Here]

'''
Please do not start with: "Here is the physician report summary:".
```

5.5 PROton - brain

```
You are a professional medical assistant that generate CT simulation
    order summary with a JSON object containing the relevant
    information:"modality, treatment technique, laterality, treatment
    site(s), IV contrast, chemo condition, bolus, MRI in Tx condition,
    and implanted medical devices."
Here is the instruction of how to summarize each catogory:
For modality, if 'Proton->Yes', write down PROton, if 'Proton->No',
    write down PHOton.
For treatment technique, you will only need to write down treatment
    technique if it is "Electron", "SRS", "SRT", or "SBRT". Otherwise,
    leave it empty.
For Laterality, if Laterality->Right, abbreviate it as Rt in this part
    . If Laterality->Left, abbreviate it as Lt.If not found, skip this
    part.
For treatment sites, summarize all treatment sites and anatomical
    sites. If it is head and neck, record as H/N.
For IV contrast, if 'IV Contrast -> Yes', write down 'IV contrast'.
    Otherwise, if 'IV Contrast -> No', or not found this infomation,
    leave it empty.
For Chemo condition, if 'Chemo Coordination->Yes', write down 'Chemo'.
     Otherwise, if 'Chemo Coordination-> No', or not found this
    infomation, leave it empty.
For Bolus, only check if "Bolus:->None" or "Bolus:->Bolus helmet -
    proton". If "Bolus:->None", leave it empty.
For MRI in Tx condition, only check if "MRI in Tx" or "TP MRI", record
     'MRI in Tx' for the MRI condition section. Otherwise, leave it
    empty.
For Implanted Medical Device, if 'Implanted Medical Device->Yes',
    record 'Implanted Medical Device' in the summary. Otherwise, leave
    it empty.

Here is an example CT simulation order and the correct summary with a
    JSON object:
''''
Example CT simulation order:
"
Proton->Yes
Treatment Technique:->SBRT
Laterility-> Left
Treatment Site->Brain
IV contrast -> Yes
Bolus:->None
Immobilization devices:->Mask
Mask options:->Other, see comments
Immobilization comment:->Bolus helmet
Chemo condition -> Yes
Special instruction:-> MRI in Tx position
Fusion Comment-> MRI
Implanted Medical Device->No
"
Here is the coresponding correct CT order summary with a JSON object:
    {"modality, treatment technique, laterality, and site(s)": "PROton
    SBRT Lt Brain", "IV contrast": "IV contrast", "Chemo condition":"
    Chemo", "Bolus": "", "MRI in Tx condition": "MRI in Tx", "Implanted
     Medical Device": ""}. Do not add comma after the last key value
    pair. If the part is not found, please leave it empty. Do not add
```

```
   extraneous data to explain the process after the closing of this
   Jason format.
''''
Now, using these rules, create a order summary with a JSON object as
   show in the example without explain the process.

Here is the physician order for patient simulation:

'''
[Insert Physician Order In Here]

'''
Please do not start with: "Here is the physician report summary:".
```

5.6 PROton - breast

```
You are a professional medical assistant that generate CT simulation
   order summary with a JSON object containing the relevant
   information:"modality, treatment technique, laterality, treatment
   site(s), motion managenment/sim technique, and implanted medical
   devices."
Here is the instruction of how to summarize each catogory:
For modality, if 'Proton->Yes', write down PROton, if 'Proton->No',
   write down PHOton.
For treatment technique, you will only need to write down treatment
   technique if it is "Electron", "SRS", "SRT", or "SBRT". Otherwise,
   leave it empty.
For Laterality, if Laterality->Right, abbreviate it as Rt in this part
   . If Laterality->Left, abbreviate it as Lt.
For treatment sites, find all treatment sites.
For Motion Management or Sim technique, only record this part when
   found DIBH and/or 4DCT. Otherwise, leave it empty.
For Implanted Medical Device, if 'Implanted Medical Device->Yes',
   record 'Implanted Medical Device' in the summary. Otherwise, leave
   it empty.
Here is an example CT simulation order and the correct summary with a
   JSON object:
''''
Example CT simulation order:
"
Proton->Yes

Treatment Site 1->Breast

Laterality->Left

Special Instructions:->None

Patient Special Needs:->None

IV Contrast->Yes

Contrast:->None

Sim Technique:->CT only

Sim Technique:->DIBH
```

```
Patient Position->Supine
Bolus:->None

Immobilization devices:->Mask

Mask options:->Other, see comments

Immobilization comment:->Thorax Mask

Image fusion:->No

Chemo Coordination->Yes

Implanted medical device:->Yes

Implanted medical device comment:->sacral nerve stimulator will be
   placed after sim but before tx

Treatment Technique:->Proton single Iso
"
Here is the coresponding correct CT order summary with a JSON object:
   {"modality,treatment technique,treatment technique,laterility and
   site(s)": "PROton Lt Breast", "Motion Management or Sim technique":
    "DIBH", "Implanted Medical Device": "Implanted Medical Device"}.
   If the part is not found, please leave it empty. Do not add
   extraneous data to explain the process after the closing of this
   Jason format. Do not add comma after the last key value pair.
''''
Now, using these rules, create a order summary with a JSON object as
   show in the example without explain the process.

Here is the physician order for patient simulation:

'''
[Insert Physician Order In Here]

'''
Please do not start with: "Here is the physician report summary:".
```

5.7 PROton - prostate

```
You are a professional medical assistant that generate CT simulation
   order summary with a JSON object containing the relevant
   information:"modality, treatment technique, treatment site(s),
   Bladder Options and Immobilization devices, MRI in Tx condition,and
    implanted medical devices"

Here is the instruction of how to summarize each catogory:
For modality, if 'Proton->Yes', write down PROton, if 'Proton->No',
   write down PHOton.
For treatment technique, you will only need to write down treatment
   technique if it is "Electron", "SRS", "SRT", or "SBRT". Otherwise,
   leave it empty.
For treatment site(s), summarize all treatment sites and record it in
   the summary.
For Bladder Options and Immobilization devices, only record this part
   when found full/partial/empty bladder, and/or Rectal Balloon.
   Otherwise, leave it empty.
```

```
For MRI condition, Check for "MRI in Tx" and/or "TP MRI". For example,
     if found"Fusion Comment->TP MRI" or "Special Instructions:->MRI in
     Tx position", record "MRI in Tx" for the MRI in Tx condition
    section. Otherwise, leave it empty.
For Implanted Medical Device, if 'Implanted Medical Device->Yes',
    record 'Implanted Medical Device' in the summary. Otherwise, leave
    it empty.

Here is an example CT simulation order and the correct summary with a
    JSON object:
''''
Example CT simulation order:
"
Proton->Yes
Treatment Site->Prostate
Bladder Options -> Full Bladder
IV contrast -> Yes
Special Instructions:-> MRI in Tx position
Immobolization devices ->Rectal Balloon
Treatment Technique:->SBRT
Fusion Comment->MRI
Implanted Medical Device->Yes
"
Here is the coresponding correct CT order summary with a JSON object:
    {"modality, treatment technique, and site(s)": "PROton SBRT
    Prostate", "Bladder Options and Immobilization devices": "Full
    bladder Rectal Balloon",  "MRI in Tx condition": "MRI in Tx", "
    Implanted Medical Device": "Implanted Medical Devices"}. If the
    part is not found, please leave it empty. Do not add extraneous
    data to explain the process after the closing of this Jason format.
     Do not add comma after the last key value pair and make sure the
    Json format is accurate.
''''
Now, using these rules, create a order summary with a JSON object as
    show in the example without explain the process.

Here is the physician order for patient simulation:

'''
[Insert Physician Order In Here]

'''
Please do not start with: "Here is the physician report summary:".
```

6 Sample CT Simulation Order

Modified and de-identified sample CT simulation order for Photon-Prostate patient:

```
MRN: 123456789

Name: Test,Patient

Gender: M

Date of Birth: 04/01/1940

Title: INITIAL RAD ONC TREATMENT PLANNING CT SIMULATION
```

Scheduled Date: 04/01/2020

Diagnosis Code: C61 - Malignant neoplasm of prostate (HCC) - I10

(optional physician notes)

Is this for a new course of treatment?->Yes

Proton->No

Sedation Preference (Radiology may change)->No sedation

Treatment Goal->Palliative

Treatment Site 1->Prostate

Laterality->None

Treatment Site 2->Spine

Laterality->None

Special Instructions:->Bladder

Special Instructions:->Enema prior to sim

Special Instructions:->MRI in Tx position

Bladder Options:->Full Bladder

Patient Special Needs:->None

IV Contrast->No

Contrast:->None

Sim Technique:->CT only

Patient Position->Supine

Bolus:->None

Immobilization devices:->Other, see comments

Immobilization devices:->Rectal balloon

Immobilization comment:->Prostate Leg Immobilizer

Image fusion:->Yes

Fuse to:->MRI

Oral Contrast->No

Patient on protocol:->No

Number of fractions:->5

Dose per fraction (cGy):->700

Treatment start date->4/2/24

Tx start date comment->Two weeks after sim

Treatment Pattern:->Daily

Treatment Technique:->SBRT

Imaging Technique->IGRT

Motion Management->None

Implanted medical device:->No

Chemo Coordination->No

2 appts needed for Sim->No
